# Whole-genome sequencing of copy number variation analysis in Ethiopian cattle reveals adaptations to diverse environments

**DOI:** 10.1186/s12864-024-10936-5

**Published:** 2024-11-15

**Authors:** Wondossen Ayalew, Wu Xiaoyun, Getinet Mekuriaw Tarekegn, Tesfaye Sisay Tessema, Min Chu, Chunnian Liang, Rakan Naboulsi, Renaud Van Damme, Erik Bongcam-Rudloff, Yan Ping

**Affiliations:** 1 Key Laboratory of Animal Genetics and Breeding on Tibetan Plateau, Ministry of Agriculture and Rural Affairs, Lanzhou, 30050 P.R. China; 2 Key Laboratory of Yak Breeding Engineering, Lanzhou Institute of Husbandry and Pharmaceutical Sciences, Chinese Academy of Agricultural Sciences, Lanzhou, 730050 P.R. China; 3 Institute of Biotechnology, Addis Ababa University, P.O. Box 1176, Addis Ababa, Ethiopia; 4 State Key Laboratory of Swine and Poultry Breeding Industry, National Engineering Research Center for Breeding Swine Industry, Guangdong Provincial Key Lab of Agro-Animal Genomics and Molecular Breeding, College of Animal Science, South China Agricultural University, Guangzhou, 510642 China; 5 Scotland’s Rural College (SRUC), Roslin Institute Building, University of Edinburgh, Edinburgh, EH25 9RG UK; 6 Childhood Cancer Research Unit, Department of Women’s and Children’s Health, Karolinska Institute, Tomtebodavägen 18A, Stockholm, 17177 Sweden; 7 Department of Animal Biosciences, Swedish University of Agricultural Sciences, Uppsala, 75007 Sweden; 8 Institute of Western Agriculture, The Chinese Academy of Agricultural Sciences, Changji, 831100 P.R. China

**Keywords:** Adaptation, Copy number variation, Ethiopian cattle, Whole-genome sequencing

## Abstract

**Background:**

Genomic structural variations (GSVs), notably copy number variations (CNVs), significantly shape genetic diversity and facilitate adaptation in cattle populations. Despite their importance, the genome-wide characterization of CNVs in indigenous Ethiopian cattle breeds—Abigar, Fellata, and Gojjam-Highland remains largely unexplored. In this study, we applied a read-depth approach to whole genome sequencing (WGS) data to conduct the first comprehensive analysis of CNVs in these populations.

**Results:**

We identified 3,893 CNV regions (CNVRs) covering 19.15 Mb (0.71% of the cattle genome). These CNVRs ranged from 1.60 kb to 488.0 kb, with an average size of 4.92 kb. These CNVRs included deletions (1713), duplications (1929), and mixed events (251) showing notable differences in distribution among the breeds. Four out of five randomly selected CNVRs were successfully validated using real time polymerase chain reaction (qPCR). Further analyses identified candidate genes associated with high-altitude adaptation (*GBE1* and *SOD1*), heat stress adaptation (*HSPA13*,* DNAJC18*, and *DNAJC8*) and resistance to tick infestations (*BoLA* and *KRT33A*). In addition, variance stabilizing transformation (*V*_*ST*_*)* statistics highlighted population-specific CNVRs, emphasizing the unique genetic signatures of high-altitude adaptation in the Gojjam-Highland cattle breed. Among the detected CNVRs, 4.93% (192 out of 3,893) overlapped with 520 quantitative traits loci (QTLs) associated with six economically important trait categories suggesting that these CNVRs may significantly contribute to the genetic variation underlying these traits.

**Conclusions:**

Our comprehensive analysis reveals significant CNVRs associated with key adaptive traits in Ethiopian cattle breeds highlighting their genetic diversity and resilience. These findings offer valuable insights into the genetic basis of adaptability and can inform sustainable breeding practices and conservation efforts. Future research should prioritize the functional validation of these CNVRs and their integration into breeding programs to enhance traits such as disease resistance and environmental adaptability.

**Supplementary Information:**

The online version contains supplementary material available at 10.1186/s12864-024-10936-5.

## Introduction

Genomic structural variations (GSVs) are large-scale DNA sequence alterations significantly contributing to genetic and phenotypic diversity [1]. Copy number variations (CNVs) are major structural alterations that involve insertions, duplications, and deletions of genomic segments ranging from 50 kilobase pairs (kbp) to several megabases [2]. While CNVs are less frequent than small insertions and deletions (InDels) and single nucleotide polymorphisms (SNPs) at the genomic level, they cover larger genomic regions and have profound functional and evolutionary impacts [3]. CNVs can modify gene structure, dosage, and regulation, contributing to adaptive evolution, diversity, and breed differentiation [4–6]. Additionally, CNVs are associated with quantitative trait loci that influence production traits, body measurements, and parasite resistance in cattle populations [7–9]. Therefore, genome-wide detection of CNVs provides valuable insights into genomic variations, facilitating the development of breeding strategies for sustainable cattle production.

In various species, including cattle, previous studies have explored CNVs using array-based platforms such as comparative genomic hybridization arrays [10, 11] and the Illumina BeadChip [7, 12, 13]. However, these approaches have limitations, including low probe density, limited genome coverage, low resolution and hybridization noise [14, 15]. Moreover, the detection of small copy number variations (CNVs) (< 10 kb) and precise identification of CNV boundaries are other limitations of array-based methodologies [16]. With the advancements in high throughput sequencing (HTS) and associated analysis programs, new strategies have emerged for systematically identifying CNVs at a genome-wide level. These sequence-based approaches have gained popularity due to ongoing developments and cost reductions in NGS technology, enabling CNV reconstruction with improved resolution and sensitivity [17–19].

The indigenous cattle populations in Ethiopia possess a rich genetic diversity owing to their long history of adaptation to various ecological and environmental conditions. Abigar, Fellata, and Gojjam-Highland cattle breeds are notable examples of indigenous cattle known for their resilience and unique adaptations to the challenging landscapes of Ethiopia. The Abigar cattle fall under the classification of “Niloitic Sanga.” They are primarily situated in the warm and humid climate of the Gambella Regional State in Ethiopia, extending to the border of South Sudan [20]. Fellata cattle are also adapted to the hot and humid lowlands of Northwestern Ethiopia’s Quara district and are believed to have originated in West Africa [21, 22]. This breed has been dispersed across west to east and Central Africa under various names, including Fulani (Nigeria), Djafoun (Cameroon), and Fellata (Chad and Ethiopia) [23, 24]. In contrast, Gojjam-Highland cattle thrive in high-altitude environments, exhibiting small body size, with an estimated mature body weight of 220.83 ± 1.28 kg [25]. This notable distinction in altitude and adaptation underscores the remarkable diversity within Ethiopian cattle populations, emphasizing the necessity to study and preserve these unique genetic resources.

Previous researches have investigated the population genetics of Ethiopian cattle populations using single nucleotide polymorphisms (SNPs) [26–29]. However, the role of CNVs in shaping these populations’ genetic and phenotypic diversity remains largely unexplored. Understanding the impact of CNVs on breed differentiation, adaptation to local environments, and economically important traits can unlock potential avenues for sustainable breeding practices and contribute to the conservation and utilization of these valuable genetic resources. In this study, we performed the whole genome characterization of CNVs in three indigenous Ethiopian cattle populations using the read depth (RD) approach. We identified important genomic regions harboring adaptation traits essential for future conservation and improvement efforts of three native cattle breeds.

## Materials and methods

### Animals and whole-genome sequencing

**Fig. 1:**
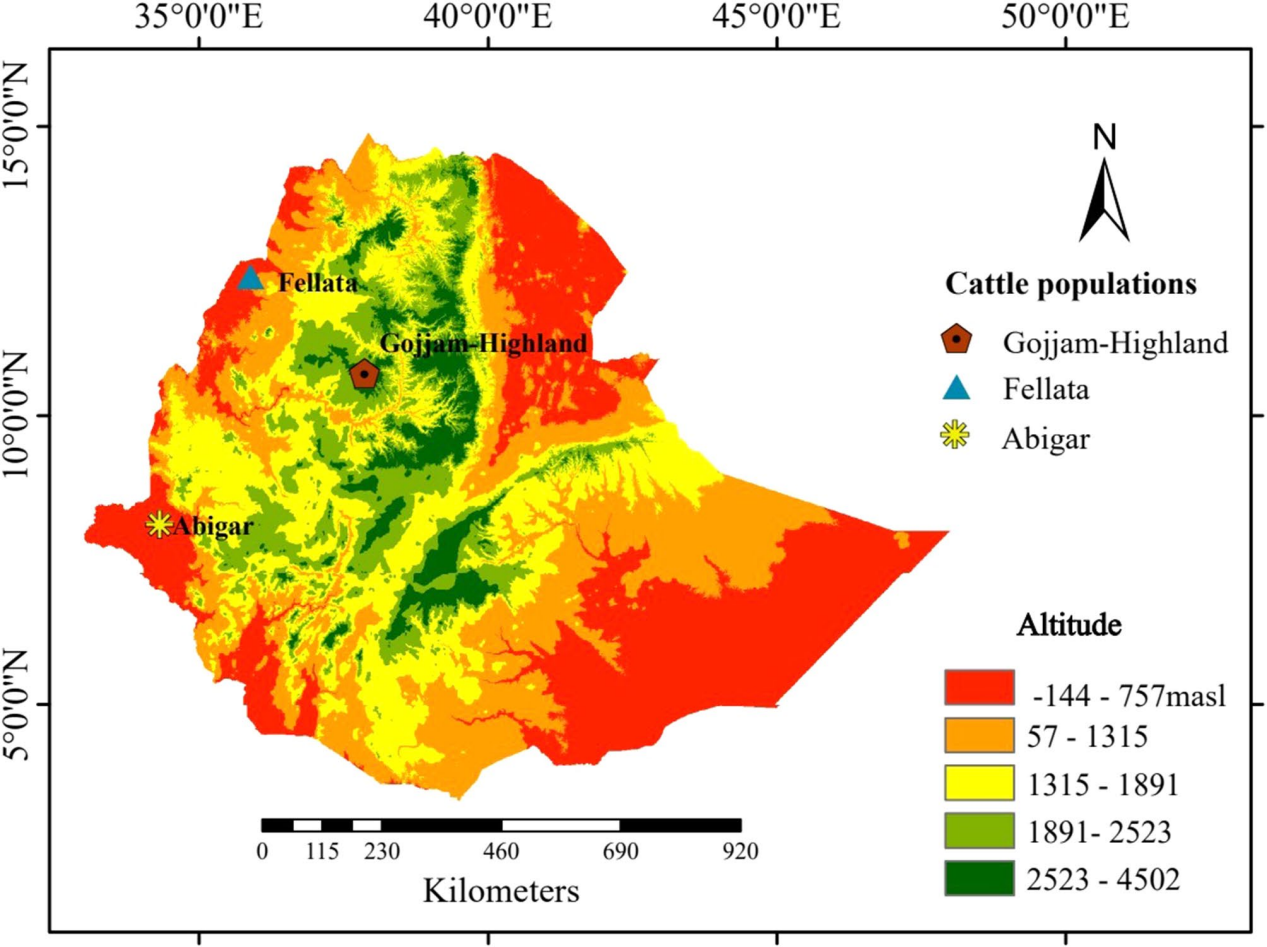
Sampling locations of the three cattle breeds

Blood samples were collected from 30 cattle from the three distinct breeds. Specifically, 10 samples were obtained from Gojjam-Highland cattle in the Choqe Mountain region of East Gojjam at 4,000 m above sea level. Additionally, 10 samples were collected from Abigar cattle in the Akobo district and 10 from Fellata cattle in the Quara district, both cattle breeds are found in the lowland regions of Ethiopia where the altitude is less than or equal to 550 m.a.s.l. (Fig. 1) [30]. Blood samples were collected aseptically from the animals without anesthesia to minimize stress and avoid harm. The procedure adhered strictly to sterile techniques, with blood drawn from the jugular vein using sterile needles and syringes, ensuring minimal discomfort. All procedures were performed by trained personnel following established ethical animal care guidelines, and no animals were euthanized at any point during the study. Blood samples were collected in the study area after obtaining informed consent from each cattle owner. Genomic DNA was extracted using the Tiangen genomic DNA extraction kit following the manufacturer’s protocols (https://en.tiangen.com/content/details_43_4224.html). Standard quality assessments were conducted on the DNA extracts followed by sequencing performed by Frasergen Bioinformatics Co., Ltd. (Wuhan, China) using the MGI-SEQ 2000 platform.

## Quality control and read alignment

We checked the quality of the raw reads using FastQC v0.11.8 [31]. Trimmomatic software v0.39 [32] was employed to remove adaptor sequences and low-quality bases using default parameters. The cleaned reads were aligned to the cattle reference genome ARS-UCD1.2 using BWA v0.7.13-r1126 [33] with default parameters. The resultant alignment files in sequence alignment map (SAM) format were sorted, indexed, and converted to the binary alignment map (BAM) format using sequence alignment/map tools (SAMtools v1.6) [34]. The Picard v2.27.4 MarkDuplicates function (https://broadinstitute.github.io/picard/) was employed to identify and account for duplicate reads. We subsequently used the resultant marked BAM files as input for CNV calling.

## Detection of copy number variations

Copy number variations (CNVs) were detected using the read depth-based method implemented in CNVcaller v0.92 [35], a tool designed for detecting CNVs from whole-genome sequencing (WGS) data. The read depth approach involves segmenting the reference genome into overlapping sliding windows of 800 bp (-w: 800 bp), and then normalizing read depth by correcting for GC content biases across the genome. In the first step, the reference genome was indexed to generate a reference database, after which CNVcaller calculated the GC-corrected normalized read depth for each sample. The CNVs were called using the default CNVcaller thresholds: a lower limit of normalized read depth set at 0.2 (-l: 0.2), an upper limit of 0.7 (-u: 0.7), and a minimum GC content of 0.5 (-g: 0.5) [36]. To eliminate redundant or overlapping calls and enhance confidence, CNVRs of all samples were merged with the default parameters using the distance between the two initial calls being less than 20% of their combined length and the Pearson’s correlation index of the two CNVRs being significant at the 0.01 level [37]. Additionally, we removed CNVs in unplaced scaffolds. For final data curation, we considered only the CNVR found in more than two samples for further analysis [37, 38]. We performed functional annotation of CNVRs using ANNOVAR [39], classifying them into intronic, exonic, or intergenic regions.

## Selective sweep analysis of CNVRs

To identify population-specific CNVs among distinct cattle breeds living in high altitudes (Gojjam-highland) and low altitudes (Abigar and Fellata) cattle breeds, we computed *V*_*ST*_ statistics (a method analogous to Wright’s fixation index, *F*_*ST*_). Following the approach outlined by Redon et al. [40], we calculated the pairwise *V*_*ST*_ as *V*_*ST*_ = (*V*_*T*_ − *V*_*S*_)/*V*_*T*_, where *V*_*T*_ is the total variance of copy numbers among two populations, and *V*_*S*_ is the average variance within each population, weighted for population size. Functional enrichment analysis was conducted on the CNVRs with the top 5% *V*_*ST*_ values as a significance threshold to identify candidate CNVRs.

## Validation of CNVRs by qPCR

To validate the identified CNVs from WGS data using CNVcaller, we performed quantitative real-time PCR using LightCycler96. Five CNVRs were randomly selected based on the CNV type (loss, gain, and both gain/loss) and frequency [41]. The primers (Additional file 8: Table S8) were designed using the Primer-BLAST web tool (https://www.ncbi.nlm.nih.gov/tools/primer-blast/index.cgi?LINK_LOC=BlastHome) based on ARS-UCD1.2 genome assembly. For each CNVR, three samples with copy number variations and one standard sample were selected based on CNVcaller software results. To establish a baseline, the bovine basic transcription factor 3 gene (BTF3) was utilized as the reference gene [41]. The qPCR was performed using Promega’s GoTaq^®^ qPCR Master Mix kit. Three replicates per sample and one standard sample for each CNVR were used. The qPCR analysis was conducted in a total reaction volume of 20 µL. The procedure included an initial pre-incubation step at 95 °C for 30 s to activate the polymerase enzyme. This reaction was followed by a two-step amplification process of 45 cycles: denaturation at 95 °C for 5 s and annealing/extension at 60 °C for 30 s. Subsequently, a melting curve analysis was conducted to assess the specificity of the amplified products. The melting curve program involved an initial denaturation step at 95 °C for 5 s, followed by annealing at 65 °C for 60 s, and a final denaturation at 95 °C for 1 s. Fold changes were calculated using the 2^−ΔΔCt^ method, and the copy number of the target genes in the test samples was determined as 2 × 2^−ΔΔCt^.

### QTLs overlapping with CNVRs and cross-validation with previous studies

To explore potential associations between CNVRs and economically significant traits in cattle, we utilized the Animal QTL database (https://www.animalgenome.org/cgi-bin/QTLdb/BT/index) to align the Cattle QTL loci with these CNVRs. Furthermore, to validate the credibility of our results, we compared CNVR counts and lengths from our study with recent publications across various cattle breeds. Ensuring consistency in genomic references, CNVRs previously mapped to the UMD 3.1 genome were converted to the ARS-UCD1.2 assembly using the LiftOver tool (https://genome.ucsc.edu/cgi-bin/hgLiftOver), with all location data annotated accordingly [42].

## Functional annotation of CNVR-harboring genes

A list of genes in the latest cattle genome assembly (ARS-UCD1.2) was downloaded from the Ensembl database, and the coordinates of CNVRs were overlapped with the intersect function of Bedtools version 2.31 [43]. Only genes that partially or entirely overlapped with CNVRs were retained for further analysis. The gene ontology (GO) and pathway (KEGG) analyses were performed using the Database for Annotation, Visualization, and Integrated Discovery (DAVID v6.8, https://david.ncifcrf.gov/) [44]. *P*-values were adjusted for multiple comparisons using the false discovery rate (*FDR*) method, with an *FDR*-adjusted *p*-value cut-off of 0.05. This adjustment is essential to control for the expected proportion of false positives in multiple testing scenarios, thereby reducing the risk of identifying spurious results.

## Results

The reliability of CNVs identified through short-read alignment depends significantly on coverage and sequencing depth. Sequence alignment metrics, including mapping rate, coverage, and mean depth, were assessed for all samples, and a comprehensive summary is presented in Additional file 1: Table S1. This analysis sequenced 791.6 Gb of sequences with an average depth of 15X. After initial quality checks, sequencing reads exhibited a mean coverage of 98.45, indicating robust depth at specific genome positions. The mean mapping percentage was 93.6%, signifying comprehensive coverage across 93.6% of the cattle reference genome (ARS-UCD1.2). These metrics affirm the data’s sufficiency and quality for subsequent CNV analysis.

**Table 1:** CNVR summary for each cattle breed

Breeds	Sample	CNVRs	Length(bp)	Unique	Dup	Del	Both	%^a^
Abigar	10	3417	17,248,400	546	1319	1848	250	0.6351
Fellata	10	3755	17,956,000	233	1668	1837	250	0.66116
GojjamH	10	3431	17,680,400	631	1360	1824	247	0.65101
Merged	30	3893	19,148,800	0	1713	1929	251	0.70508

^a^ Percentage of total CNVR length in the reference genome (ARS-UCD1.2); GojjamH = Gojjam-Highland

### Number and distribution of CNVRs

**Fig. 2:**
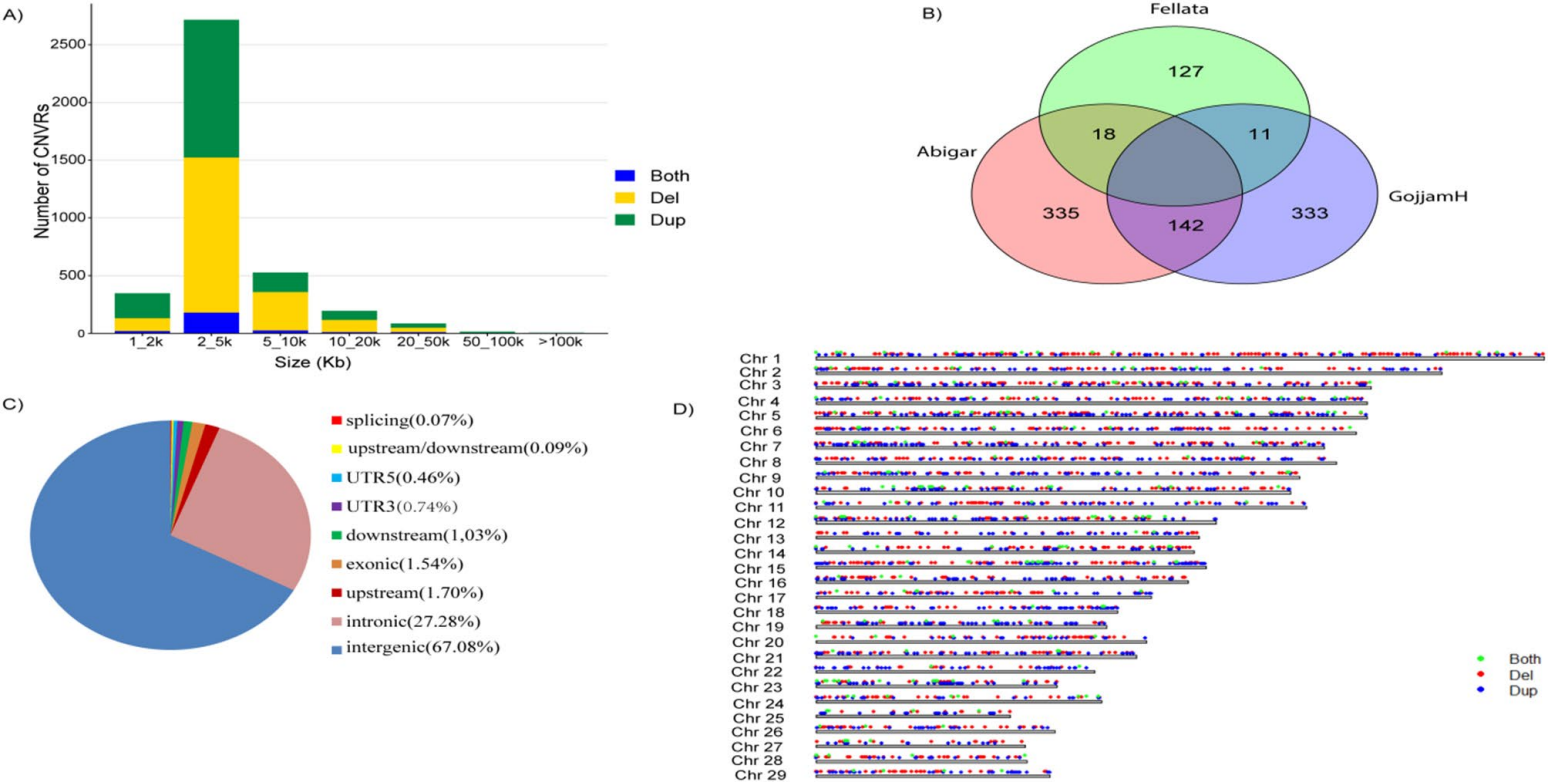
The genomic distribution of Copy Number Variation Regions (CNVRs) (**a**) The count of detected CNVRs (**b**) Annotations of CNVRs with various genomic features (**c**) Venn diagram depicting the numbers of CNVRs identified in three distinct Ethiopian cattle breeds (**d**) The autosomal distribution of CNVRs across cattle autosomes

After quality control, we identified 3,893 CNVRs by consolidating overlapping CNVRs into unique regions with overlaps identified in at least two animals. The CNVRs collectively covered a length of 19.15 Mb, accounting for approximately 0.71% of the cattle genome (ARS-UCD1.2). The CNVRs comprised 1,929 deletions, 1,713 duplications, and 251 mixed-type events (containing deletions and duplications) (Table 1). In terms of size distribution, the analysis revealed that 69.77% of CNVRs fell within the 2–5 kb range, 13.54% within the 5–10 kb range, and 8.94% within the 1–2 kb range, with the remaining exceeding 10 kb in size (Fig. 2a). The Venn diagram demonstrated a lack of overlap of CNVRs among all three populations. Specifically, 335 CNVRs were uniquely distributed in Abigar, 127 CNVRs were exclusive to Fellata, and 333 CNVRs were unique to Gojjam-Highland cattle breeds (Fig. 1b). Functional classification indicated that 2,612 CNVRs (67.09%) were in intergenic regions, while 1,065 CNVRs (27.36%) were within intronic regions. Only 60 (1.54%) CNVRs were identified within coding exonic regions (Fig. 2c). Furthermore, the distribution of these CNVRs across chromosomes was non-uniform (Fig. 2d).

### Comparison of CNVR results with existing studies

Our study compared CNVRs identified in cattle with those reported in recent studies, as summarized in Table 2. The total number of CNVRs in the referenced studies varied from 923 to 18,391, with total CNVR lengths ranging from 4.66 Mb (0.162% of the cattle genome) to 287.74 Mb (10.66%). Our investigation revealed overlapping CNVRs with some previous studies, with overlap rates ranging from 2.57 to 65.73%. The lengths of these overlapping segments varied from 100 Kb to 2.559 Mb (Table 2).

**Table 2:** Comparison of CNVRs with recent reports in cattle

Study	Studied breed	Number of breeds	Number of Animals	Reference genome used	Sequencing /genotyping platform	Number of CNVRs	Mean size of CNVR (kb)	Total length of CNVRs (Mb)	Number of overlapping CNVRs	Ratio of overlapping CNVRs (%)
Upadhyay et al., [45]	Multi breeds	38	196	UMD3.1	Illumina BovineHD	923	66.15	61.06	210	5.40
Strillacci et al., [46]	Multi breeds	3	108	UMD3.1	Illumina BovineHD	1723	34.50	59.44	211	5.42
Liu et al., [11]	Holsteins	1	47	UMD3.0	CGH	976	36.13	35.26	122	3.13
Lee et al., [47]	Multi breeds	3	475	ARS-UCD1.2	Illumina BovineHD	1755	39.67	69.62	438	11.25
Hu et al., [48]	Multi breeds	8	73	ARS-UCD1.2	Illumina high-Seq	13,234	3.06	40.53	1196	30.72
Jang et al., [38]	Multi breeds	39	336	ARS-UCD1.2	Illumina high-Seq	18,391	15.65	287.74	2559	65.73
Sun et al., [49]	Simmental	1	30	ARS-UCD1.2	BGISEQ-500	2944	1.583	4.66	100	2.57
This study	Multi breeds	3	30	ARS-UCD1.2	MGI-SEQ 2000	3893	4.92	19.15	-	-

### Validation of CNVRs by qPCR

**Fig. 3:**
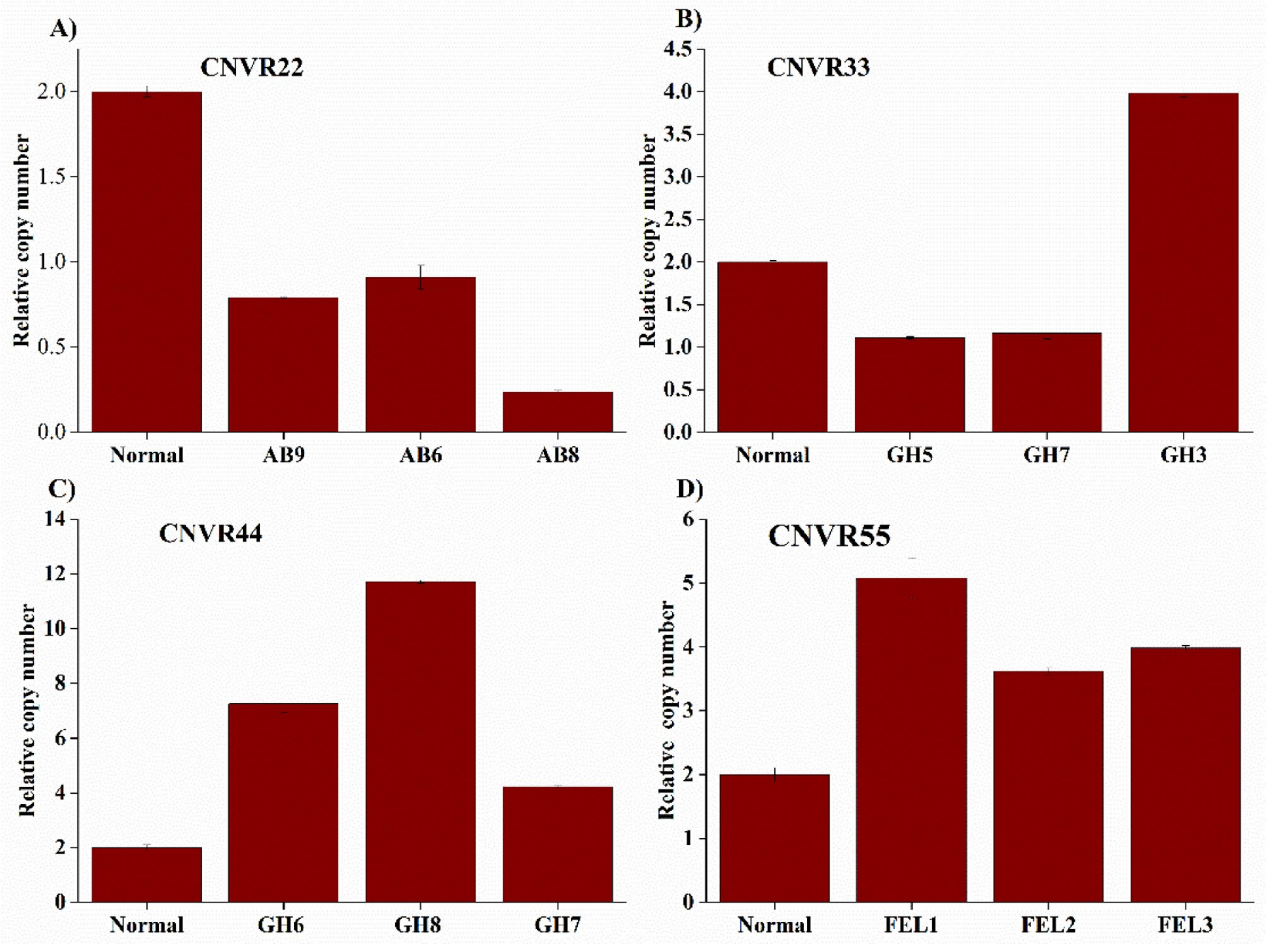
The qPCR results for three validated CNVRs. The y-axis indicates the normalized ratios, while the x-axis denotes the references and samples (AB = Abigar; GH = Gojjam-Highland; FEL = Fellata). Samples with normalized ratios around 0 or 1 indicate instances of copy number loss, while those with values around three or higher suggest copy number gains (*P* < 0.05). A value of 2 signifies a normal copy number

Five CNVRs were randomly selected to validate the consistency of our CNVRs detected from WGS data using CNVcaller. Among the five regions, four of these CNVRs (80%) showed consistent copy number states (loss, normal, and gain) between qPCR and whole-genome sequencing (Fig. 3a, b,c, d). The first CNVR identified was CNVR11, categorized as a duplication-type CNV. However, the qPCR analysis revealed no evidence of copy number duplication in the tested samples.

### Functional annotations and enrichment analysis

**Fig. 4:**
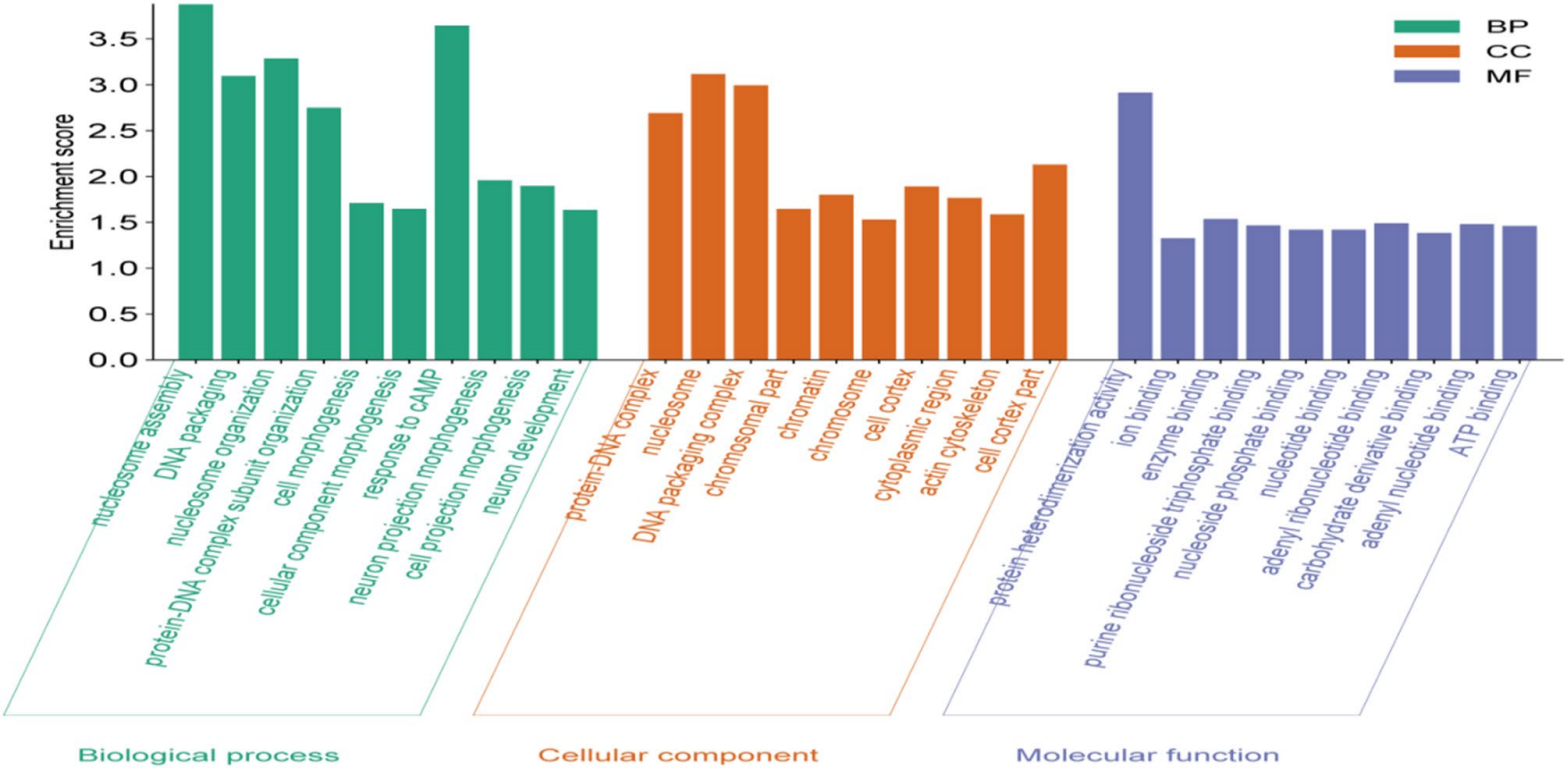
The top ten significant gene ontology terms enriched in CNVR-harbor genes

**Fig. 5:**
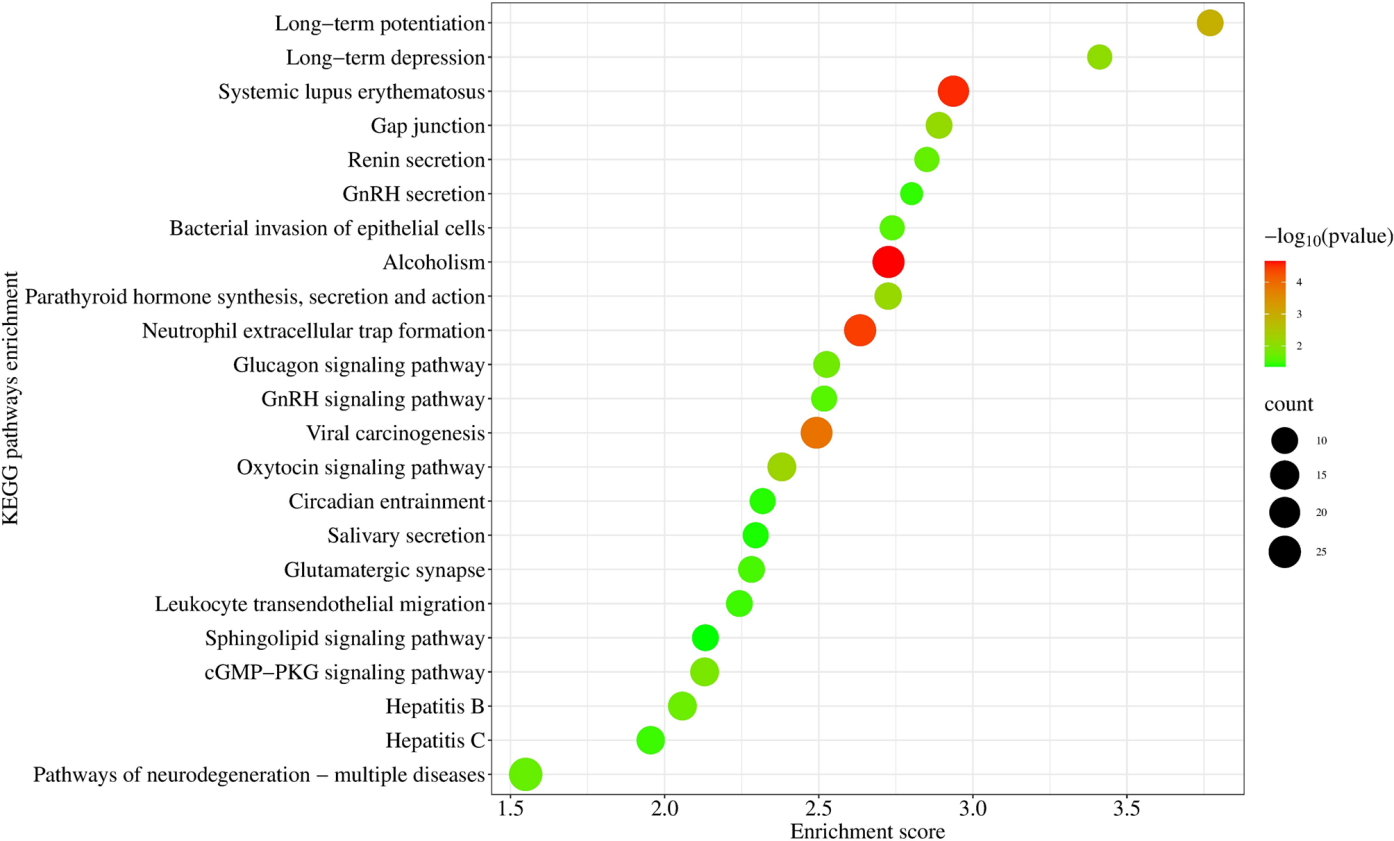
The KEGG pathways enriched in CNVR-harbor genes

Of the 3893 identified CNVRs, 1082 (27.8%) CNVRs either resided within or partially overlapped with the cattle genome, covering 792 protein-coding genes (Additional file 2: Table S2). The analysis of protein-coding genes revealed 166 significant GO terms (Additional file 3: Table S3) and 23 KEGG pathways showing enrichment (Additional file 4: Table S4). Notably, the identified significant GO terms encompass diverse biological functions; cell morphogenesis, response to cAMP, and cellular component morphogenesis emerged as the top three functional terms. The enriched GO terms associated with genes located within CNVRs were systematically categorized based on biological process, cellular component, and molecular function, and their rankings are illustrated in Fig. 4. Furthermore, specific genes related to renal secretion, bacterial invasion of epithelial cells, and circadian entrainment were enriched in KEGG pathways (Fig. 5). Furthermore, the biological roles of several CNVR-harbored genes were identified to be associated with high-altitude adaptations (*GBE1 and SOD1*), heat stress adaptation (*HSPA13*,* DNAJC18*, and *DNAJC8*), and resistance to tick infestations (*BoLA* and *SLC25A48*) (Additional file 2: Table S2).

### Differential CNVRs in ethiopian cattle

**Fig. 6:**
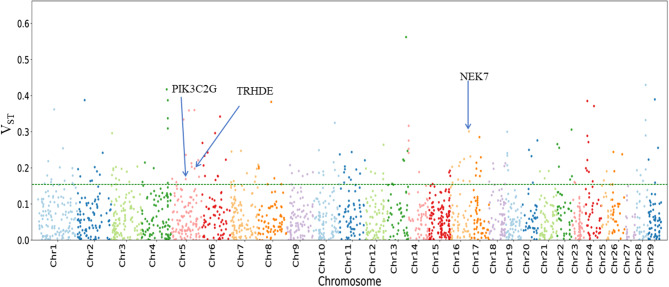
Manhattan plot for *V*_*ST*_ analysis between high altitudes (GojjamH) and low altitude (Abigar and Fellata) cattle breeds (top 5%)

To compare the CNVRs of Ethiopian cattle inhabiting high-altitude regions with those living in low-altitude areas, we performed *V*_*ST*_ statistics. The upper 5% threshold in the *V*_*ST*_ test revealed a total of 195 CNVRs that overlapped with 71 genes. After excluding hypothetical, putative, predicted, or uncharacterized genes and pseudogenes, we identified 55 protein-coding genes as potential candidates (Additional file 5: Table S5). Notably, the genes *PIK3C2G* (*V*_*ST*_ score = 0.19), *NEK7* (*V*_*ST*_ score = 0.31), and *TRHDE* (*V*_*ST*_ score = 0.21) were all identified above the threshold line and exhibited significant differentiation between high and low-altitude cattle breeds, indicating their association with high-altitude adaptations in Gojjam-Highland cattle (Fig. 6). The functional annotations of these protein-coding genes enriched 82 significant GO terms (Additional file 6: Table S6).

### QTLs overlapping with identified CNVRs

To further investigate the CNVRs linked to economically important traits, the identified CNVRs were compared with QTLs from the cattle QTL database. Among the 3893 identified CNVRs, 192 (4.93%) overlapped with 520 QTLs associated with six distinct trait categories. These categories encompass diverse phenotypic traits, including reproduction, production, meat and carcass quality, milk production, exterior morphology, and health attributes. Specifically, the distribution of QTLs across these categories was as follows: reproduction QTLs (7.11%), production QTLs (8.46%), meat and carcass QTLs (24.23%), milk QTLs (62.50%), exterior QTLs (5.96%), and health QTLs (3.85%) (Additional file 7: Table S7).

## Discussion

Copy number variations are crucial contributors to genetic diversity, influencing phenotypic variations in livestock species. Genome-wide detection of CNVs is essential for understanding the complex genomic landscape and unraveling their implications for economically significant traits and evolutionary processes. Among the various methods for CNV elucidation, the RD approach stands out as a highly accurate and widely adopted technique in current CNV analysis. Ensuring sufficient coverage and sequencing depth of short-read data is imperative for the robust identification of CNVs [38]. Ethiopia boasts a diverse reservoir of cattle genetic resources and is subject to various environmental conditions, resulting in varying performances among its cattle populations. This inherent diversity renders Ethiopian cattle particularly conducive to CNV studies. Nevertheless, despite these favorable circumstances, Ethiopia has a noticeable shortage of CNV research. This study addresses this gap by embarking on the first genome-wide CNV detection effort in three Ethiopian cattle breeds (Abigar, Fellata, and Gojjam-Highland) employing WGS data.

### CNVRs detected in the cattle genome

Our analysis identified 3,893 CNVRs spanning approximately 19.15 Mb, constituting around 0.71% of the cattle genome. These CNVRs exhibited a diverse size range, ranging from 1.60 kb to 488.0 kb, with an average size of 4.92 kb. In addition, we detected more deletion events than duplication, which is consistent with reported results in American mink [50] and Tibetan sheep genome [51]. This could be due to the fact that read-depth analysis tends to be more sensitive to detecting missing reads (deletions) compared to identifying additional reads (duplications).To validate our CNVR predictions, we conducted qPCR assays on a subset of CNVRs. Encouragingly, four of five CNVRs were confirmed to align with CNVcaller predictions. However, the first CNVR (CNVR11) validation yielded unsuccessful results, possibly due to issues with the qPCR primer design. It is conceivable that the primer design inadvertently targeted regions outside the true CNVR, resulting in an inaccurate assessment of copy number variation [16, 41]. This underscores the complexity of CNV detection and emphasizes the limitations of computational algorithms, such as CNVcaller, in accurately characterizing CNVRs.

To build upon our validation efforts, we compared the CNVRs identified in our study with those reported in recent literature, as detailed in Table 2. Several key differences emerged in the number of CNVRs identified and their total lengths, highlighting the diversity and complexity of genomic variations across different breeds and study methodologies. In our study, we identified 3,893 CNVRs spanning a combined length of 19.15 Mb. This result contrasts with other studies using the ARS-UCD1.2 reference genome. For instance, Jang et al. [38], who analyzed a larger sample size using the Illumina high-Seq platform, identified a notably higher number of CNVRs, totaling 18,391, with a total length of 287.74 Mb. Similarly, Hu et al. [48] reported 13,234 CNVRs totaling 40.53 Mb in multi-breed cattle using the same platform. In contrast, Sun et al. [49], focusing on a single breed (Simmental) and utilizing the BGISEQ-500 platform, identified 2,944 CNVRs with a notably smaller total length of 4.66 Mb. These variations suggest that breed-specific genetic architectures and sequencing platforms can yield different CNVR profiles. Factors such as sample size, sequencing platform, genome coverage, sequencing depth, and detection algorithms likely influence CNVR detection [19, 52]. Additionally, studies employing the Illumina BovineHD array based on the UMD3.1 reference genome, including Upadhyay et al. [45] and Strillacci et al. [46], reported fewer CNVRs (923 and 1,723, respectively) but with larger mean sizes, resulting in total lengths of 61.06 Mb and 59.44 Mb, respectively. This discrepancy with our findings may be due to differences in the resolution and sensitivity of detection platforms. Higher-density arrays, such as the Illumina BovineHD, tend to detect larger CNVRs with greater precision but may miss smaller variations due to probe distribution and coverage limitations. In contrast, whole-genome sequencing (WGS) provides higher resolution and can identify smaller CNVs that arrays might miss [15, 17]. Furthermore, variations in reference genome assemblies can impact CNV detection, as different assemblies may vary in their completeness and accuracy in representing genomic regions [53, 54].

### CNVR functional enrichment: insights into molecular adaptations to environmental stresses

Researchers widely acknowledge that Ethiopian cattle breeds demonstrate remarkable adaptability to diverse ecological zones, exhibiting resilience against various challenges such as diseases, parasites, feed shortages, and harsh environmental conditions [55, 56]. The results of GO and KEGG enrichment analyses suggest that CNVs may play a role in critical biological processes related to immunity, including the bacterial invasion of epithelial cells and adaptations such as neuron development and cell morphogenesis. In particular, bacterial invasion of epithelial cells has been implicated in triggering inflammatory responses, representing a pivotal mechanism for clearing infecting microbes [57]. Moreover, studies have demonstrated that gene ontology (GO) enrichment analyses of specific genes uncover functional terms associated with cell morphogenesis under stress conditions, such as heat shock stress [58]. The intricate interplay of genetic factors, particularly CNVs, in regulating processes like cell morphogenesis emphasizes the dynamic nature of the molecular mechanisms underlying the adaptability of Ethiopian cattle breeds, especially in response to heat stress.

In this study, GO enrichment and KEGG analyses identified several essential genes. We found *GBE1* and *SOD1* associated with hypoxia at high altitudes among these. The *GBE1* gene (CNVR_Chr1:29.10–29.41 Mb) encodes the enzyme glycogen branching enzyme 1, which is essential for glycogen metabolism, particularly for the synthesis and branching of glycogen [59]. Under hypoxic conditions, *GBE1* undergoes up-regulation through the hypoxia-inducible factor-1 (*HIF1*) signaling pathway [60], inducing a metabolic shift from oxidative phosphorylation to glycolysis and promoting increased glycogen synthesis. This process results in the accumulation of glycogen, a crucial storage form of glucose in the body [61], suggesting the potential significance of *GBE1* in high-altitude adaptation. *SOD1* (*CNVR_Chr1:38.24–38.33Mb*) is a crucial antioxidant enzyme targeting superoxide radicals. Hypoxia, characterized by reduced oxygen availability, induces an imbalance in cellular redox status, accumulating reactive oxygen species (*ROS*), including superoxide radicals [62]. *SOD1* plays a vital role in counteracting oxidative stress by catalyzing the dismutation of superoxide radicals into oxygen and hydrogen peroxide [63]. This enzymatic activity contributes to the restoration of redox balance, mitigating the harmful effects of ROS. Notably, *SOD1* is known to activate ATP production [64] and reduce mitochondrial ROS production [65]. Positive selection of this gene in hypoxia adaptations has been elucidated in Tibetan sheep [66], emphasizing *SOD1*’s role as a crucial antioxidant player during hypoxic conditions. This underscores its significance in maintaining cellular homeostasis and offers potential implications for therapeutic interventions in hypoxia-related disorders.

The escalating impact of climate change, characterized by rising environmental temperatures and prolonged droughts, substantially influences livestock health and welfare. In light of these challenges, exploring candidate genes crucial for heat stress adaptations becomes a pivotal avenue for understanding and addressing the evolving needs of livestock. Our study identified candidate genes associated with heat stress adaptations linked to cattle breeds inhabiting hot and low-altitude environments, notably the *HSPA13* and *Hsp40* Protein family (*DNAJC18* and *DNAJC8*) genes. *HSPA13* (*CNVR_Chr1:22.78–22.794Mb*) gene belongs to the *HSP70* family and is renowned for its multifaceted involvement in various cellular stress responses. Its functions extend beyond molecular chaperoning, playing a pivotal role in protein folding [67, 68]. The upregulation of *HSPA13* in response to heat stress underscores its significance in fostering cellular adaptation and survival under elevated temperatures [69]. This dynamic response aligns with the broader functions of the *HSP70* family, recognized for their versatility in mitigating diverse stressors within the cellular environment.

Similarly, East African Shorthorn Zebu cattle harbor a member of the heat shock protein family (*DNAJC18* and *DNAJC18*) that responds to heat stress [70]. These findings suggest that identifying heat stress-related candidate genes in indigenous cattle, such as Abigar and Fellata cattle breeds, could elucidate the genetic mechanisms behind their adaptation to humid and hot environments. Identifying heat-responsive genes in diverse cattle breeds contributes to our understanding of their adaptive strategies, which is imperative for devising targeted interventions to boost resilience against climate-induced challenges. Our study revealed genes within CNVRs associated with immune system processes, including those related to tick resistance (*BoLA* and *KRT33A*) (Additional file 2: Table S2). The bovine lymphocyte antigen (*BoLA*) gene is a crucial element of the histocompatibility complex, playing a pivotal role in the intricate antigen processing and presentation processes. Recent studies by Kim et al. [26] and Taye et al. [55] have emphasized the substantial contribution of the *BoLA* gene to regulating the immune response, particularly concerning its role in conferring tick resistance in African cattle. The *KRT33A* gene is a constituent of the keratin gene family that encodes proteins involved in synthesizing intermediate filaments. These filaments are crucial in furnishing structural support to skin and hair cells. Furthermore, their involvement in tick resistance becomes apparent as they act as a protective barrier against the external environment [71]. Additionally, keratinocytes within the epidermis exhibit immune response functions by secreting cytokines that trigger local inflammatory responses [71].

To identify significant CNVRs showing divergence between distinct populations, we calculated pairwise *V*_*ST*_ values between high-altitude (Gojjam-Highland) and low-altitude (Abigar and Fellata) cattle breeds. Our analysis revealed three CNVRs containing the *NEK7*,* PIK3C2G*, and *TRHDE* genes, which exhibited significant differentiation between high and low-altitude cattle breeds. The *NEK7* gene, also known as NIMA (never in mitosis gene A)-related kinase 7, encodes a protein belonging to the *NEK* family of serine/threonine protein kinases [72]. Serine/threonine kinases, such as the AMP-activated protein kinase (*AMPK*) family, play a crucial role in signaling pathways that interact with the hypoxia-inducible factor (*HIF*) pathway [73]. *AMPK* activation facilitates energy conservation, aids adaptation to low oxygen levels, and stabilizes *HIF-1α*, a pivotal player in the transcriptional response to hypoxia [74]. Additionally, *NEK7* activates the *NLRP3* inflammasome [75], and identifying *NLRP3* upregulation as a critical component in the cellular response to hypoxia suggests its potential contribution to cellular adaptation and survival under conditions of oxygen deprivation and energy crisis [76]. The two noteworthy candidate genes, *PIK3C2G* and *TRHDE*, play roles in hypoxia adaptations in Tibetan and Ethiopian sheep [77, 78]. However, the specific roles of *PIK3C2G* and *TRHDE* in the adaptation to high altitudes remain speculative. The presence of these genes in the Gojjam-Highland breed likely impacts critical adaptive traits such as metabolic efficiency, oxygen transport, and stress management. Understanding these contributions could lead to targeted breeding strategies aimed at enhancing these traits, thereby improving performance and resilience in high-altitude environments. Future research, including transcriptome analyses and functional validations, is imperative to elucidate the actual contributions of these genes in Ethiopian cattle. Such investigations will provide deeper insights into the genetic mechanisms underpinning environmental adaptation and inform strategies for optimizing the resilience of Ethiopian cattle.

### Association between CNVRs and QTLs for economic traits in cattle

The overlap between CNVRs and QTLs in cattle reveals significant insights into genetic architecture, aiding the understanding of crucial traits and informing marker-assisted selection and genomic prediction. We identified 192 CNVRs overlapping with 520 QTLs across six trait categories, highlighting the importance of considering structural variants in genomic studies to enhance cattle breeding programs. These categories include reproduction, production, meat and carcass quality, milk production, exterior morphology, and health attributes (Additional file 7: Table S7). Our findings are consistent with prior research, which has demonstrated that CNVRs often contain QTLs linked to economically significant traits in cattle [9, 38, 46, 49].

Disease resistance and milk production are essential traits of Ethiopian cattle. Among the identified QTLs related to these traits, tick resistance and milk yield are particularly important for improving cattle productivity and health. One of the promising candidate genes identified within a QTL associated with tick resistance is *GIMAP8*. This gene belongs to the *GTPase* family of immunity-associated proteins (*GIMAPs*) and is prominently expressed during the terminal stages of T and B cell development [79]. It plays a critical role in the maturation and function of these immune cells. Robbertse et al. [80] highlighted that in bovine regions affected by tick infestations, there is a significant influx of B lymphocytes and a notable increase in CD3 + T lymphocytes, particularly in tick-resistant breeds. This indicates that B lymphocytes are pivotal mediators of the immune response in these cattle. In this study, the identified QTL loci included *CSN1S1* and *PRKG2* as potential candidate genes for milk production. *CSN1S1*, encoding alpha-s1 casein, plays a crucial role in milk production by influencing milk composition [81, 82]. Its genetic variants affect milk yield and quality, making it a valuable target for selective breeding programs aimed at enhancing dairy productivity [82]. *CSN1S1* allelic diversity is associated with variations in milk protein content and cheese-making traits, underscoring its significance in the dairy industry [83]. The application of genomic technologies provides valuable insights into the molecular mechanisms underlying both adaptive and productive traits, paving the way for significant advancements in selective breeding strategies for dairy cattle.

## Conclusions

This study presents a comprehensive genome-wide analysis of copy number variations (CNVs) in three indigenous Ethiopian cattle populations, revealing significant genetic diversity and distinct CNV patterns associated with adaptive traits. The results demonstrate that CNVs play a crucial role in the genetic adaptation of these breeds to their environments, particularly in relation to high altitude, heat stress, and tick resistance. The key genes linked to these adaptive traits provide valuable insights into the molecular mechanisms underlying resilience in Ethiopian cattle populations. However, the relatively small sample size may affect the robustness of our findings. Further studies are required to investigate these mechanisms across a broader range of cattle populations utilizing larger sample sizes. Despite this limitation, this study improves our understanding of genetic variation in Ethiopian cattle and provides a basis for future breeding and conservation strategies. Functional validation of the identified CNVs and their associated genes will be essential to translate these findings into practical applications for improving the resilience and productivity of cattle.

## Electronic supplementary material

Below is the link to the electronic supplementary material.

Supplementary Material 1

## Data Availability

The Ethiopian indigenous cattle pair-end raw sequencing data (in fastq.gz format) were available at NCBI under the bio-project accession number PRJNA1053488 (https://www.ncbi.nlm.nih.gov/bioproject/?term=PRJNA1053488) and PRJNA1059514 (https://www.ncbi.nlm.nih.gov/bioproject/1059514).

## Abbreviations

BAF: B allele frequency
BAM: Binary alignment map
CGH: Comparative genomic hybridization
CNV: Copy number variation
CNVR: Copy number variation region
DAVID: Database for annotation, visualization, and integrated discovery
FST: Fixation index
FDR: False discovery rate
GSVs: Genomic structural variations
GO: Gene ontology
HTS: High throughput sequencing
KEGG: Kyoto encyclopedia of genes and genomes
qPCR: Real-time quantitative polymerase chain reaction
QTL: Quantitative trait locus
RD: Read-depth
SNP: Single nucleotide polymorphisms
SAMtools: Sequence alignment/map tools
VST: Variance stabilizing transformation
WGS: Whole genome sequencing
